# Low-frequency pulsed electromagnetic fields significantly improve time of closure and proliferation of human tendon fibroblasts

**DOI:** 10.1186/2047-783X-19-37

**Published:** 2014-07-05

**Authors:** Claudine Seeliger, Karsten Falldorf, Jens Sachtleben, Martijn van Griensven

**Affiliations:** 1 Department of Trauma Surgery, Experimental Trauma Surgery, Klinikum rechts der Isar, Technical University Munich, Ismaninger Strasse 22, D-81675 Munich, Germany; 2 Sachtleben GmbH, Falkenried 88, 20251 Hamburg, Germany

**Keywords:** wound healing, proliferation, apoptosis, low-frequency pulsed electromagnetic fields

## Abstract

**Background:**

The promotion of the healing process following musculoskeletal injuries comprises growth factor signalling, migration, proliferation and apoptosis of cells. If these processes could be modulated, the healing of tendon tissue may be markedly enhanced. Here, we report the use of the Somagen™ device, which is certified for medical use according to European laws. It generates low-frequency pulsed electromagnetic fields that trigger effects of a nature that are yet to be determined.

**Methods:**

A 1.5-cm wide, linear scrape was introduced into patellar tendon fibroblast cultures (N = 5 donors). Treatment was carried out every second day. The regimen was applied three times in total with 30 minutes comprising pulsed electromagnetic field packages with two fundamental frequencies (10 minutes of 33 Hz, 20 minutes of 7.8 Hz). Control cells remained untreated. All samples were analyzed for gap closure time, proliferation and apoptosis one week after induction of the scrape wound.

**Results:**

The mean time for bridging the gap in the nontreated cells was 5.05 ± 0.33 days, and in treated cells, it took 3.35 ± 0.38 days (*P* <0.001). For cell cultures with scrape wounds, a mean value for BrdU incorporation of OD = 0.70 ± 0.16 was found. Whereas low-frequency pulsed electromagnetic fields treated samples showed OD = 1.58 ± 0.24 (*P* <0.001). However, the percentage of apoptotic cells did not differ between the two groups.

**Conclusions:**

Our data demonstrate that low-frequency pulsed electromagnetic fields emitted by the Somagen™ device influences the *in vitro* wound healing of patellar tendon fibroblasts and, therefore, possibly increases wound healing potential.

## Background

One of the most important advances in promotion of the healing process following musculoskeletal injuries has evolved from the insight that treatment of these injuries with prolonged immobilization may delay recovery and adversely affect normal tissues. Conversely, controlled early resumption of activity can promote restoration of function. Experimental studies in the several past decades confirm and help explain the deleterious effects of prolonged immobilization and the beneficial effects of activity on the musculoskeletal tissues [1,2]. At the beginning of the healing process, controlled motion and loading of tendon and ligament repair tissue help align the regeneration of cells and collagen fibers, stimulate collagen synthesis and increase strength [3-6]. Early or excessive strain, however, can increase the inflammatory reaction and may damage repair tissue, leading to failure of the healing process [7].

However, not only mechanical loading or growth factor signalling is important for healing processes. DNA activity concerning transcription and translation, as well as cell cycle mechanisms, plays a pivotal role. Those activities comprise proliferation, migration and apoptosis of cells. If these processes could be modulated, the healing of tendon tissue may be enhanced markedly. This modulation could prevent the occurrence of excessive strain by accelerating tendon healing.

In order to study such processes *in vitro*, wound-healing assays have been carried out in tissue cultures for many years. These assays monitored cell behavior, including appraising the migration and proliferative capacities of different cells under various culture conditions. They generally involve growing cells to a confluent monolayer as a first step. The layer is ‘wounded’ by a scraping device (razor-blade, pipette tip, needle or cell-scraper). This penning in the cell layer gets repopulated because the cells on the wound edge are no longer contact-inhibited. At the cellular level, healing involves the cells’ detachment from and attachment to the matrix adjacent to the wound area, migration, and proliferation. This repopulation is microscopically observed over a time course to assess the gap closure time, the occupied area over time, or the rate of migration [8-10]. Moreover, proliferation and apoptosis are investigated regularly. Depending on the cell type, the growth factors present, and the extent of the wounded region, wound repair ranges from several hours to days.

Until the 1980s it was believed that biological information within cell systems was being transferred not only chemically but also physically via electromagnetic waves. Information of this nature activates or inhibits biochemical processes [11,12].

**Figure 1:**
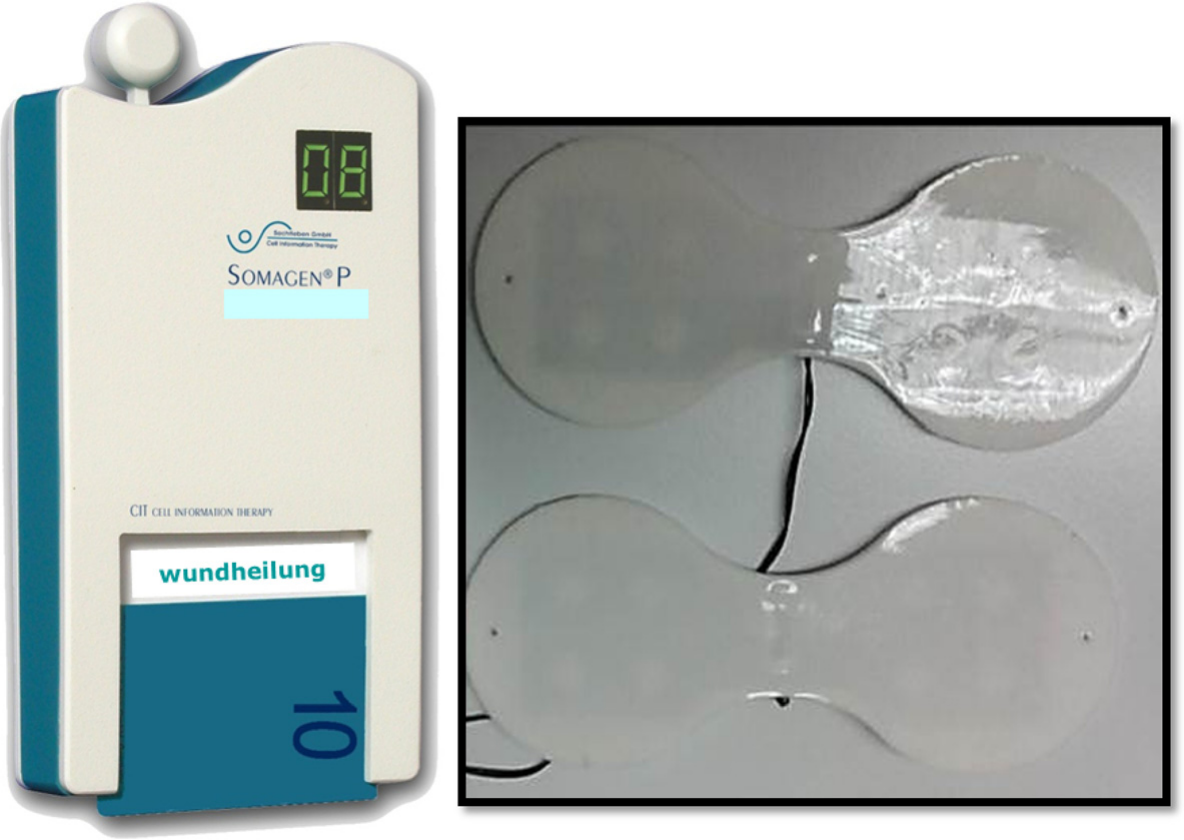
**The low-frequency pulsed electromagnetic fields (PEMF) emitting Somagen™ device.** In this work, a specific ‘wound healing’ program lasting 30 minutes was used. The applied program consisted of two PEMF signal packages of 10 minutes at a fundamental frequency of 33 Hz and 20 minutes at 7.8 Hz.

Led by these findings in the early 1990s, Sachtleben GmbH, Hamburg, Germany developed the Somagen™ device, which supposedly stimulates the communication mechanisms of cells (Figure 1). The low-frequency pulsed electromagnetic fields (PEMF) electromagnetic signals have been described as affecting enzymes, cells, tissues and whole organisms. Even though the effects exerted by PEMF could be measured, the reasons for the reactions of the biological systems remain unidentified. However, several theories exist to explain these effects, for example the Larmor precession [13,14], the hypothesis of Gartzke and Lange [15] or radical pair mechanism [16-18] (for review see [19]). The application of the PEMF induces changes in cellular processes, among others, differentiation [20], apoptosis [21], DNA synthesis [22], protein expression [23], protein phosphorylation [24], anti-inflammatory effects [25] and hormone production [26].

PEMF instruments like the Somagen™ device generate low-frequency electromagnetic signals in order to accelerate, among others, wound healing response. This enhances the regeneration potential of the destroyed tissue, especially the stimulation of new formation of connective tissue, something for which the vasodilatation and increased cell division are likely responsible [27]. Furthermore, growth factor signalling, which is important for healing processes, can be influenced by low-frequency electromagnetic signals. Zhao *et al*. could demonstrate a stimulation of the VEGF receptor signaling pathway by applying an electric field on vascular endothelial cells [28]. Another study demonstrated an increased type I collagen expression in fibroblasts after exposure to pulsing electric fields [29]. Zhao *et al*. summarized that electric fields polarize the activation of multiple signalling pathways, including the PI3 kinases/Pten, membrane growth factor receptors and integrins, both key players in the wound healing processes [30].

However, the effect of low-frequency PEMF emitted by the Somagen™ device on fibroblasts as key players in wound healing remains to be investigated. Therefore, this study focuses on the effects of PEMF on the healing process of tendon fibroblasts in an *in vitro* wound healing model. Our findings may be helpful in the field of ligament tissue engineering and may support the development of new strategies for ligament repair.

## Methods

### Cell culture

Fibroblasts were isolated from five patients undergoing surgical treatment of the knee joint. The study protocol is in accordance with the standards of the Declaration of Helsinki. Following approval by the ethical committee of Hannover Medical School, written informed consent was obtained from the patients. The specimens of approximately 4 × 2 mm were aseptically collected from the patellar tendon. The obtained patellar tendon specimen was divided into 0.5 mm^2^ pieces and transferred into petri dishes with a roughened bottom. Dulbecco’s Modified Eagle’s Medium (DMEM) was used as culture medium containing 10% fetal calf serum, 1% gentamicin and 1% amphotericin B (Biochrom, Berlin, Germany). Tissue specimens were cultured in a humidified environment with 5% CO_2_ at 37°C. Medium was replenished every second day. After six to eight days, fibroblasts started to grow out of the patellar tendon specimens. After another three to four weeks, the cells reached 80 to 90% confluence. The cells were trypsinized and subcultured in 75 cm^2^ flasks (13 × 10^3^ cells/cm^2^). Concomitantly, they were counted and an overall viability of more than 90% was observed using the trypan blue exclusion test. This procedure was repeated once. Cells in the second passage were harvested and 1.5 × 10^5^ fibroblasts were transferred into six-well tissue culture plates (Corning, Vienna, Austria).

### Induction of the scrape wound

Scrape wounds were performed in confluent monolayer cultures of the patellar tendon fibroblasts. A 1.5 cm wide, linear scrape was introduced with a cell scraper over the entire diameter of the well. The wound area was marked with three black ink dots on each side of the wound for reference. Cultures were rinsed with culture medium to remove floating cellular debris, and fresh culture medium was added.

### Low-frequency pulsed electromagnetic fields treatment protocol

Cell cultures were treated every second day, three times in total, with a registered and certified Somagen™ device, according to company’s protocol (Sachtleben GmbH, Hamburg) In this work, a specific “wound healing” program was used. The applied program consisted of two PEMF signal packages of 10 minutes at a fundamental frequency of 33 Hz and 20 minutes at 7.8 Hz. This ‘wound healing’ program was developed by Sachtleben GmbH in cooperation with different dermatology clinics and has been successfully used before in a clinical setting [31]. The signals have the shape of spike pulses with varying send/pause intervals. Thereby, a magnetic flux density of 0.25 μT up to 3.16 μT emerged. At a 5-mm distance from the applicator, electric field strength up to 6.3 mV/cm was measurable (Additional file 1). Applicators attached to the Somagen™ device were placed in the incubator. The six-well tissue culture dishes were put directly on top of the applicators, thereby having a distance to the fibroblast monolayer of approximately 1 to 2 mm. Control cells were also put on the applicator without starting the program and were cultivated in a separate incubator to avoid interactions between the stimulated and nonstimulated cells.

In order to measure any deviation between the treated versus the control cell cultures, time to closure of the gap, proliferation and apoptosis were determined.

### Time to closure

**Figure 2:**
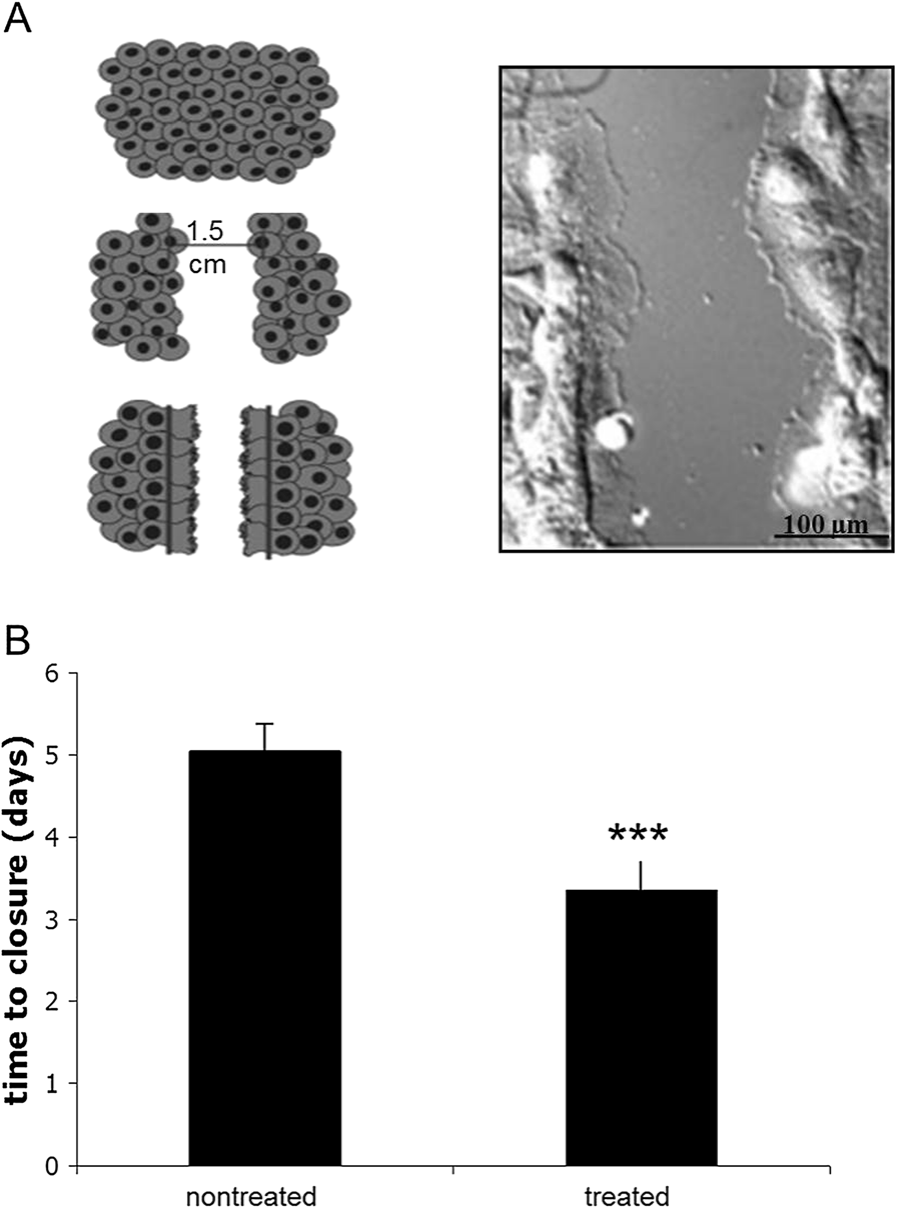
**The use of low-frequency pulsed electromagnetic fields (PEMF) lead to a significantly lower time to closure.** Scrape wound of patellar tendon fibroblasts caused by a cell scraper **(A)**, magnification 60×. For the analysis of the time to closure, one part of the cells was treated with the Somagen™ device applying the ‘wound healing’ program **(B)**. Nontreated cells were cultured under the same conditions without treatment with the Somagen™ device. Mean ± SD, ***P <0.001, (N = 5, n = 2) are indicated.

The wound was microscopically examined daily for repopulation of the wound area (Figure 2A). The end point of observation was the complete bridging of the scrape wound. Therefore, before the scratch was initiated, a photograph as control with a 20× magnification was captured with the microscope (Zeiss). Afterwards, a photograph with the same magnification was made every day. For quantification, the free area was highlighted, calculated and compared to the control with the software ImageJ 1.42q (National Institute of Health, Maryland, USA). Three independent calculations of each donor were made.

### Proliferation

The analysis of cell proliferation was performed one week after induction of the scrape wound using a standard BrdU kit for spectrophotometry (Roche, Mannheim, Germany). BrdU is a thymidine analog that is incorporated into the DNA during the synthesis phase (S1) of the cell cycle. At 0, 6 and 12 hours after application of BrdU, the amount of inserted BrdU was analyzed according to a modified protocol for the larger dishes. To remove non-incorporated BrdU, cells were washed twice with DMEM. Washed cells were fixed with 70% ethanol in 0.5 M HCl at -20°C for 30 min and washed three more times with DMEM. Nucleases were added to the cells at 37°C for 30 min to increase the accessibility of the incorporated BrdU for detection by anti-BrdU Fab-fragment. This incubation was performed in a buffer containing 66 mM Tris, 0.66 mM MgCl_2_, and 1 mM 2-mercaptoethanol to permeate the cells and disintegrate disulphide bonds. After washing the cells three times with DMEM, a mouse monoclonal Fab-fragment against BrdU conjugated with horse-radish peroxidase was added to the cells together with 10 mg/ml BSA in phosphate-buffered saline. The cells were incubated at 37°C for 30 min and subsequently washed three times with DMEM. The bound conjugate was visualized using 1 mg/ml of the soluble chromogenic substrate 2,2'-Acinobis [3-ethylbenzthiazoline-sulfonic acid] (ABTS). The signal was increased by adding 1 mg/ml of ABTS-substrate enhancer. The optical density of each sample was measured at 405 nm and 490 nm.

### Apoptosis rate

Analysis of apoptosis was performed one week after induction of the scrape wound according to the protocol provided by the manufacturer (Bender Med systems, Vienna, Austria). Briefly, adherent cells were detached from the cell culture dishes by carefully scratching with a cell scraper. The cells were centrifuged at 1500 × g and 4°C; afterwards, the pellet was carefully resuspended in 100 μl binding buffer (10 mM HEPES, pH 7.4; 140 mM NaCl; 5 mM CaCl_2_) and stained with 6 μl recombinant human annexin-V-FITC and 6 μl of propidium iodide for discrimination of living, apoptotic and necrotic cells (Bender Med Systems, Vienna, Austria). After incubation for 20 min at 4°C in darkness, the cells were centrifuged and resuspended in 100 μl binding buffer. Flow cytometry was carried out on a FACS-calibur (Becton-Dickinson, Heidelberg, Germany). The software Cellquest-pro V1.1 from Becton-Dickinson was used for data analysis.

### Statistical analysis

All experiments were performed in duplicates for each of the five patients. Furthermore, cells of each donor were divided into two groups: treated and nontreated. Data are presented as mean ± standard deviation. Differences between the treated and nontreated patellar tendon fibroblasts were analyzed using Student’s t-test. A *P* value of less than 0.05 was considered statistically significant.

## Results

### Characterization of the patellar tendon fibroblasts

Patellar tendon fibroblasts were used for cell culture. Characterization of the cells was carried out as described before [32].

### Time to closure

A uniform 1.5-cm-wide scrape wound was observed in every well of the six-well tissue culture plates. The edges of the wounds were sharply delineated. Damaged cells were observed in the edges that still adhered to the bottom of the well. On the consecutive days, the wound area was occupied by fibroblasts. The mean time for bridging the gap in the nontreated cells was 5.05 ± 0.33 days (Figure 2B). Treatment with the specific ‘wound healing’ program emitted by Somagen™ device significantly accelerated the bridging time to 3.35 ± 0.38 days (*P* <0.001).

### Apoptosis rate

**Figure 3:**
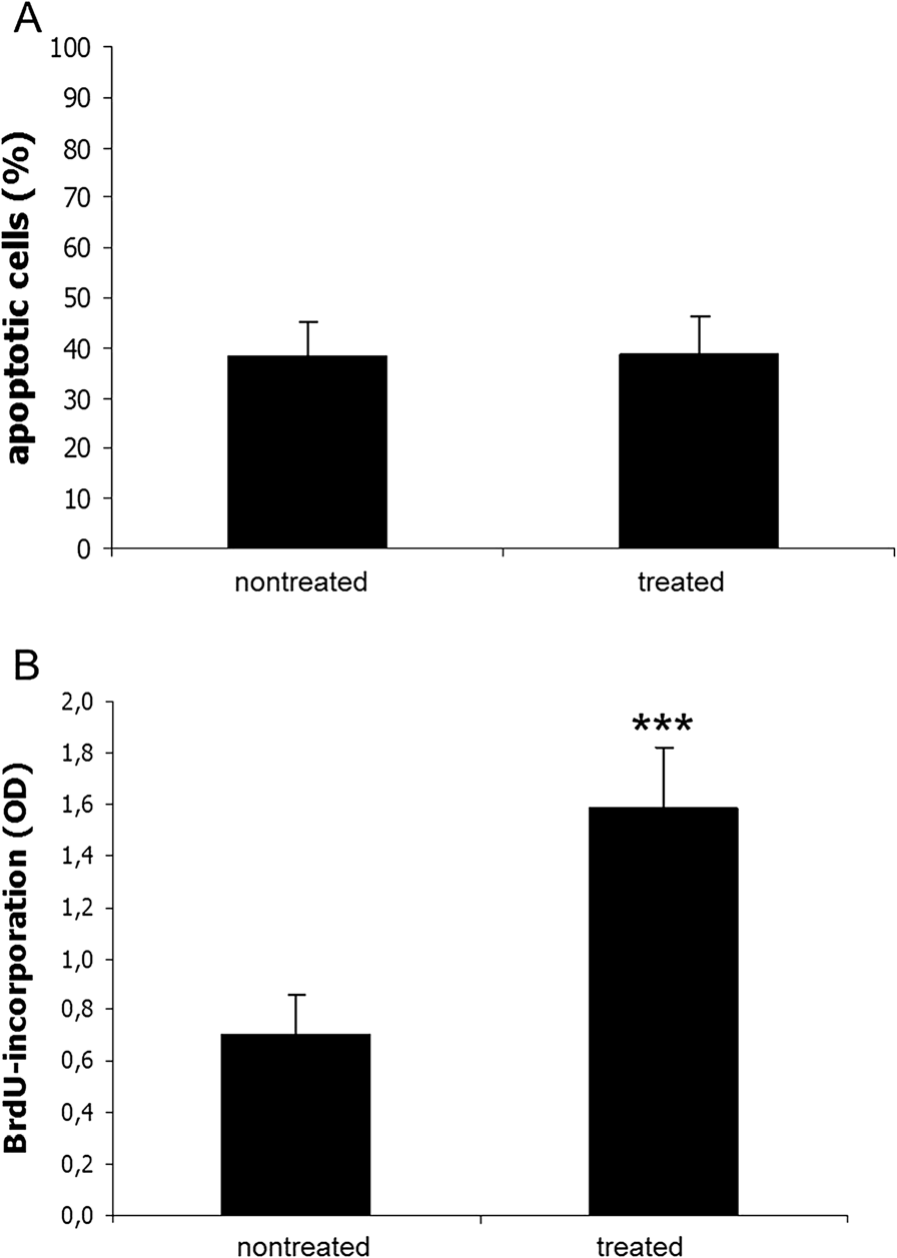
**The low-frequency pulsed electromagnetic fields (PEMF) did not affect the apoptotic rate but significantly increased the proliferation.** Apoptosis level in patellar tendon fibroblasts in the scrape wound after 1 week **(B)**. Apoptosis was measured using Annexin V-FITC and PI staining by flow cytometry. Proliferation measured using BrdU incorporation into patellar tendon fibroblasts in the scrape wound after 1 week **(A)**. One part of the cells was treated with the low-frequency PEMF generated by the Somagen™ device applying the ‘wound healing’ program. Nontreated cells served as negative control. Mean ± SD, ***P <0.001, (N = 5, n = 2) are indicated.

The percentage of Annexin-V positive cells did not differ between the two groups (nontreated 38.5 ± 6.5% versus Somagen™ device-treated 38.7 ± 7.7%) as depicted in Figure 3A.

### Proliferation

Proliferation was determined by BrdU incorporation. The obtained values are optical density values corrected for unspecific backgrounds (Figure 3B). Untreated cell cultures with scrape wounds showed a mean value of 0.70 ± 0.16. A significant increase was observed after application of the specific ‘wound healing’ program emitted by Somagen™ device (1.58 ± 0.24, *P* <0.001).

## Discussion

We investigated that certain low-frequency PEMF sequences influence *in vitro* wound healing of patellar tendon fibroblasts possibly via increasing the proliferation rate. In a similar model of scrape wounding of human foreskin fibroblasts, the 0.8-mm-wide gap was closed within 36 hours due to a preassembled matrix-containing fibrinogen. Moreover, this accelerated closure of the gap was associated with an 8-fold increase in 3H-thymidine incorporation, indicating a high proliferation rate [10]. Rodemann *et al*., who treated skin fibroblasts with electromagnetic fields, could detect a significant increase of the collagen synthesis and the protein content [33]. The proliferation capacity of the cells probably plays a role in the secondary wound healing phase. As noted in similar models using intestinal epithelial cells or endothelial cells, the rate of cell proliferation, determined by BrdU incorporation, did not differ between migrating and stationary cells over the initial 24-h period [34-36]. This indicates that early epithelial and endothelial restitution is independent of proliferation. After the migration phase that allows cells to go beyond the wound edges, cells have to proliferate in order to repopulate the wound area.

These processes are modulated by signal transduction pathways. The second messenger Ca^2+^ seems to be involved, as brief treatment with increased extracellular Ca^2+^ during scrape wounding accelerated wound area closure rates by 50% [37,38]. In our study, the tendon fibroblasts display 30% better wound area closure rates by low-frequency PEMF treatment. The differences may be due to the different cell origin, namely skin fibroblasts in the literature and tendon fibroblasts in our study. Furthermore, the multi-functional signal transducer NF-κB was activated as soon as 30 minutes after scrape wounding [35]. Especially at the wound edges, the subunit p65 was found. Within 5 minutes after wounding, ERK activation was evident. Again, this activation was particularly prominent in cells residing at the scrape edge [9]. These signal transduction molecules are important during adaptation and healing processes of tendon fibroblasts. This has been observed using cyclic, longitudinal strain in patellar tendon fibroblasts. Fifteen minutes of strain elicit NF-κB binding to DNA and is associated with increased proliferation [39,40]. c-fos and JNK are also activated [41]. Therefore, low-frequency PEMF may activate these signal transduction pathways.

These signal transduction pathways are not only involved in proliferation but also in apoptosis. In our model, 30 to 40% apoptosis of patellar tendon fibroblasts was observed. This is in concert with earlier observations using the same type of cells [41]. Treatment with the specific ‘wound healing’ low-frequency PEMF program did not result in any changes in apoptosis rates. Epithelial cells showed induction of apoptosis originating at the wound edges, but this apoptotic effect subsequently spread over a 24-hour period to encompass areas not originally damaged [42].

Our study included only five replicates; therefore, more studies are necessary to further investigate the positive effect of low-frequency PEMF in a larger cohort of samples. Additionally, *in vivo* studies should confirm these results in a whole organism with tendon pathology.

Nevertheless, the treatment with low-frequency PEMF enhances the wound healing potential of patellar tendon fibroblasts *in vitro*. The incidence of tendon and ligament injuries grows due to the increasingly sports-oriented society. Treatment of such injuries is still a challenge to orthopedic trauma surgeons as a *restitutio ad integrim* can hardly be achieved. Therefore, new modes of treatment are investigated to improve the outcome of such pathologies. Low-frequency PEMF seems to have no adverse effects when applied in the human situation [31]. Furthermore, it is non-invasive, easy to handle, and has a short application time.

## Conclusions

These results may be extrapolated to wound-healing phenomena in other soft tissues, for example skin and muscle. Wound healing is a complex process involving many different cell types and coordinated signalling responses, but fibroblasts, as a part of this complexity, support the healing process and in our study show an improved wound area closure rate under the influence of low-frequency PEMF. Thus, low-frequency electromagnetic signals could be an interesting new treatment option for wound-healing processes *in vivo* by accelerating closure of the wounds. Based on the positive results, further *in vivo* studies using low-frequency PEMF generated by the Somagen™ device for modulating wound healing are planned.

## Abbreviations

ABTS 2: 2'-Acinobis [3-ethylbenzthiazoline-sulfonic acid]; BrdU: Bromodesoxyuridin; DMEM: Dulbecco’s Modified Eagle’s Medium; FITC: fluorescein isothiocyanate; OD: optical density; PEMF: pulsed electromagnetic fields.

## Competing interests

The authors declare that they have no competing interests. Sachtleben GmbH provided the Somagen™ device for this project free of charge. Jens Sachtleben and Karsten Falldorf are both managing directors of Sachtleben GmbH.

## Authors’ contributions

CS and MvG conceived and designed the study. CS and MvG performed the experiments and analyzed the data. KF and JS provided data on the device and reviewed the manuscript. All authors read and approved the final manuscript.

## Supplementary Material

Additional file 1 Somagen™ measured field data. Click here for file
