# Supplementary material for: Low-frequency pulsed electromagnetic fields significantly improve time of closure and proliferation of human tendon fibroblasts
Source: Eur J Med Res. 2014 Jul 5;19(1):37. doi: 10.1186/2047-783X-19-37 (PMC4096547; doi:10.1186/2047-783X-19-37)
Supplement: Additional file 1 — Somagen™ measured field data. [file 2047-783X-19-37-S1.pdf]

HIGHLY CONFIDENTIAL!

Methods appear to be valid, but more detail is needed to fully understand the characteristics of the field applied.

For IP reasons we would like to reveal as little specifics as possible about applied CIT fields or programs. CIT fields operate in the extreme low frequency (ELF) range with carrier frequencies between 3,3 and 101,7 Hz.

What was the amplitude of the triangle wave? How was magnetic flux measured?

- In the presented fibroblasts-experiment the CIT program lasted 30 minutes with two different carrier frequencies applied (20 minutes of 7.8Hz and 10 minutes of 33 Hz). The signals consist of increasing spike pulses with varying send/pause intervals.

- With the setup and equipment described below an average magnetic flux density of 260 nT (along the marked segments of the applicator surface, see Figure 4-6 below) with a peak magnetic flux density 3200 nT was measured.

CONFIDENTIAL!

4.2.1 Test Equipment

Inventory No.	Test Equipment	Manufacturer	S/N	Next Calibration
30001-00007	EMI Software ES-K1	Rohde&Schwarz	V1.71	-
20001-06178	EMI Test Receiver 20Hz-26,5GHz ESU26	Rohde&Schwarz	100308	10.04.2013
20001-06144	Monitor Loop FESP 5134-40	Schwarzbeck	5134-40-054	24.01.2013

Tab. 4-3: Test equipment, radiated emission H-field

4.2.2 Setup

Vertical position of the H-field probe was 5 mm above the applicator pad measurement points. The horizontal position of the probe was centered over every measurement point.

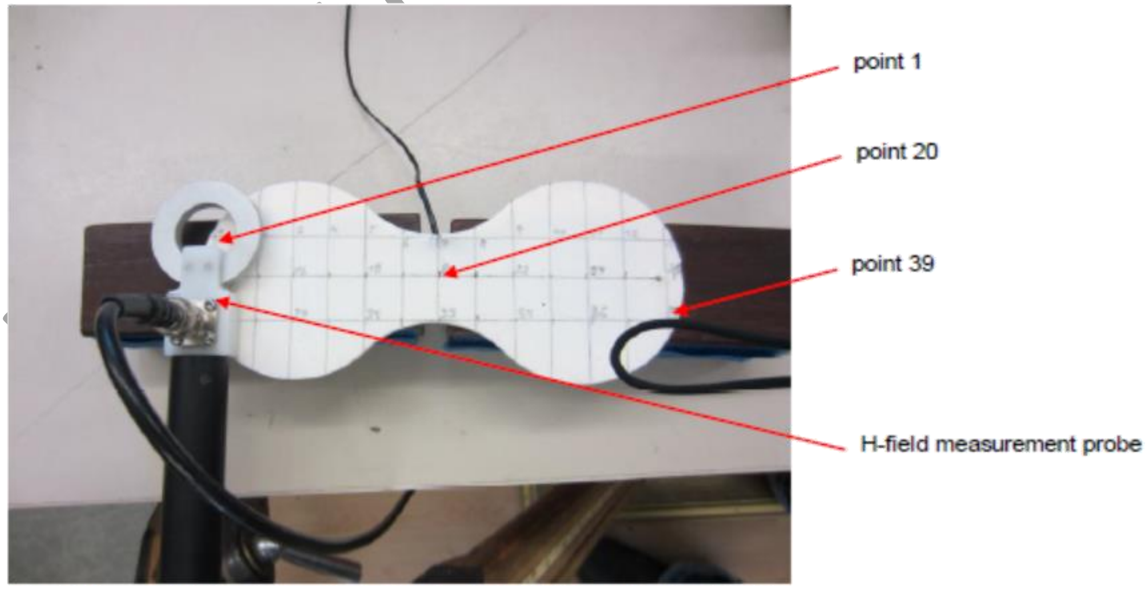

Fig. 4-6: Test setup, radiated emission H-field, marked pad and H-Field probe

How was electric field calculated, with what device? Is this a radiofrequency field?

With the setup and equipment described below a maximal electric field strength of 6.3 mV/cm was measured.

4.1.1 Test Equipment

Inventory No.	Test Equipment	Manufacturer	S/N	Next Calibration
30001-00007	EMI Software<br>ES-K1	Rohde&Schwarz	V1.71	-
20001-06178	EMI Test Receiver 20Hz-26,5GHz<br>ESU26	Rohde&Schwarz	100308	10.04.2013
20001-06018	Near Field Probe Set 7405<br>904 Ball Probe	EMCO	11414	-

Tab. 4-1: Test equipment, radiated emission E-field

4.1.2 Setup

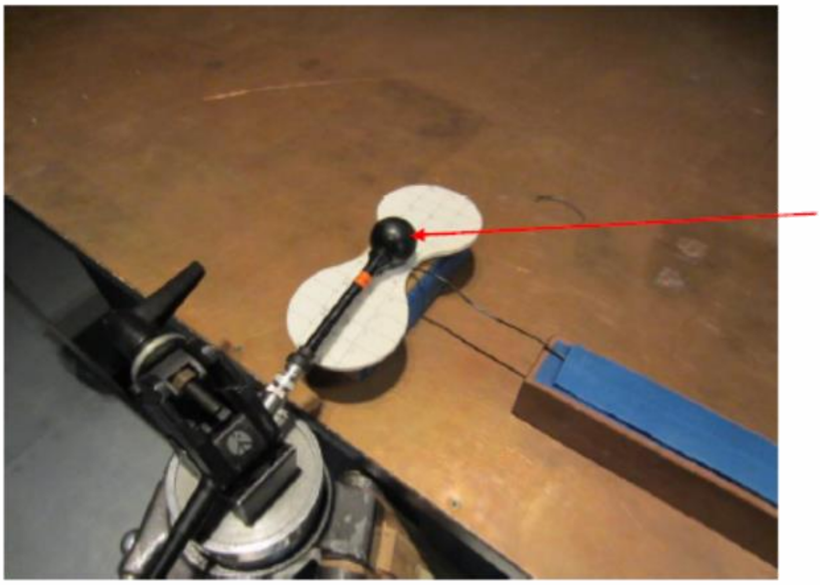

Fig. 4-1: Test setup, radiated emission E-field

As the following graph from our measurements illustrates CIT fields might emit up into the radiofrequency spectrum. However, the amplitude decreases generally with increasing frequency.

More data is available on request.

**Operating State:**	1 (card no. 96)
**Measuring Point:**	measurement point 20 of applicator
**Antenna height:**	ball probe direct above applicator
**EUT Position:**	5 cm above conductive table ground plane
**Remark:**	peak and average detector in frequency range up to 16 MHz

```
SCAN TABLE: "Sachtleben_E-Feld"

  Start      Stop       Step       Detector   Meas.     IF       Transducer
  Frequency  Frequency  Width                 Time      Bandw.
  100.0 kHz  1.0 MHz    500.0 Hz   MaxPeak    1000.0 ms 1 kHz    904_Ball_Probe
                                   Average
  1.0 MHz    100.0 MHz  5.0 kHz    MaxPeak    1000.0 ms 10 kHz   904_Ball_Probe
                                   Average
```

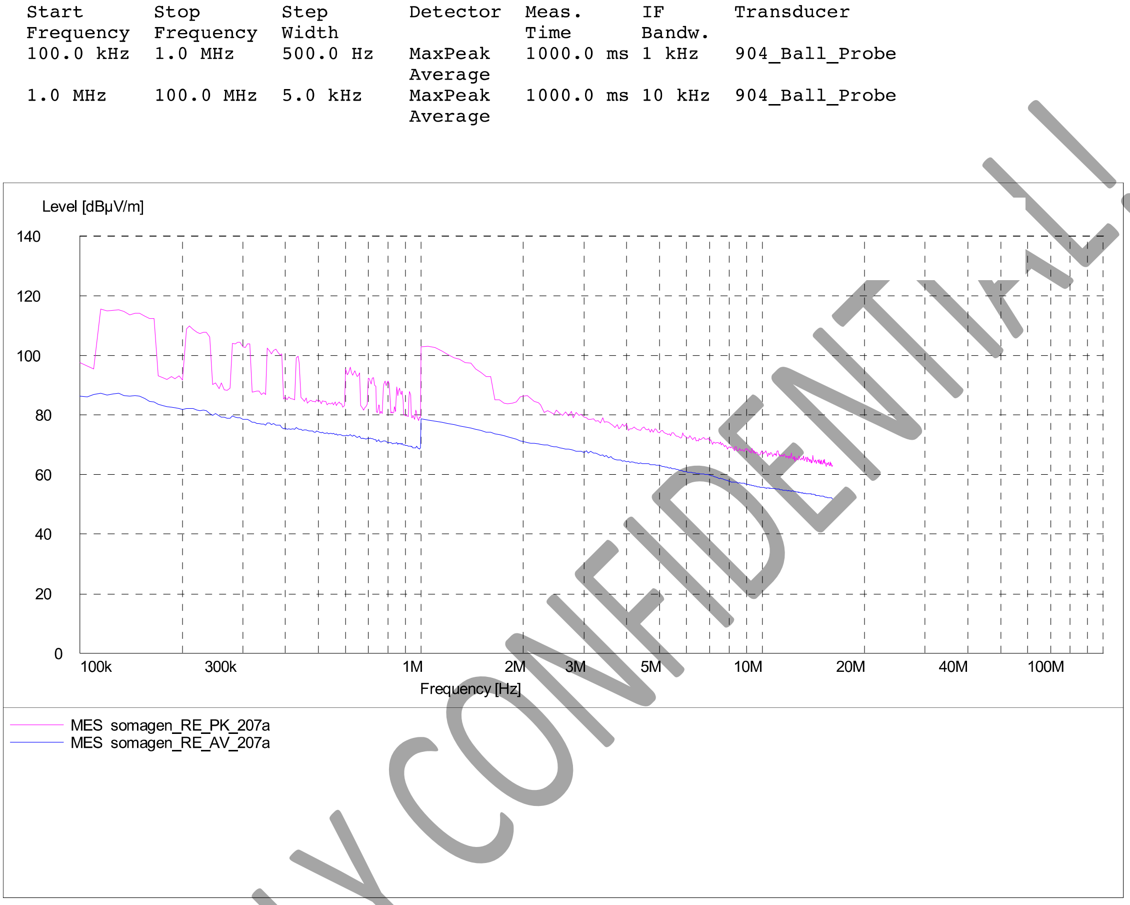

Fig. 4-4: Results, radiated emission E-field, op. state 1, peak / average detector 100 kHz - 16 MHz

HIGHLY CONFIDENTIAL!
